# Small RNA Regulation of Virulence in Pathogenic *Escherichia coli*

**DOI:** 10.3389/fcimb.2020.622202

**Published:** 2021-01-27

**Authors:** Brandon M. Sy, Jai J. Tree

**Affiliations:** School of Biotechnology and Biomolecular Sciences, University of New South Wales, Sydney, NSW, Australia

**Keywords:** sRNAs, *Escherichia coli*, post-transcriptional regulation, RNA-binding proteins, Shiga toxins, locus of enterocyte effacement

## Abstract

Enteric and extraintestinal pathotypes of *Escherichia coli* utilize a wide range of virulence factors to colonize niches within the human body. During infection, virulence factors such as adhesins, secretions systems, or toxins require precise regulation and coordination to ensure appropriate expression. Additionally, the bacteria navigate rapidly changing environments with fluctuations in pH, temperature, and nutrient levels. Enteric pathogens utilize sophisticated, interleaved systems of transcriptional and post-transcriptional regulation to sense and respond to these changes and modulate virulence gene expression. Regulatory small RNAs and RNA-binding proteins play critical roles in the post-transcriptional regulation of virulence. In this review we discuss how the mosaic genomes of *Escherichia coli* pathotypes utilize small RNA regulation to adapt to their niche and become successful human pathogens.

## Introduction

Within a few hours of birth we are colonized by our first commensal *Escherichia coli* strains. These microorganisms reside in our gastrointestinal tract and are prominent members of the gut microbiota. This association with *Escherichia coli* persists for our entire lives (Milani et al., 2017).

While a majority of *Escherichia coli* strains are commensals, some have acquired repertoires of virulence traits that allow them to thrive in unusual environmental niches and cause disease in humans. The majority of virulence factors appear to have been horizontally acquired on mobile genetic elements such as bacteriophages, transposons or plasmids, resulting in a highly mosaic genome (Rasko et al., 2008; Lukjancenko et al., 2010; Gordienko et al., 2013). Pathogenic *Escherichia coli* strains are divided into different pathotypes depending on the location of infection as well as the combination of virulence factors expressed (Kaper et al., 2004). Broadly, pathogenic *Escherichia coli* can be divided into extraintestinal pathogenic *Escherichia coli* (ExPEC) and diarrheagenic *Escherichia coli* (DEC). ExPEC cause diseases such as urinary tract infections, meningitis and sepsis and includes uropathogenic *Escherichia coli* (UPEC), sepsis-associated *Escherichia coli* (SEPEC) and neonatal meningitis *Escherichia coli* (NMEC) (Dale and Woodford, 2015). DEC pathotypes cause diarrheal diseases and are classified by the presence of characteristic virulence factors that potentiate disease. They are classified into enteropathogenic *Escherichia coli* (EPEC), enterohemorrhagic *Escherichia coli* (EHEC), enterotoxigenic *Escherichia coli* (ETEC), enteroaggregative *Escherichia coli* (EAEC), enteroinvasive *Escherichia coli* (EIEC) and diffusely adherent *Escherichia coli* (DAEC).

The virulence factors that define these pathotypes can either be integrated into the chromosome within pathogenicity islands or encoded on accessory plasmids. These include adhesins and colonization factors, toxins, and altered metabolic pathways, all of which are expressed in a coordinated manner to facilitate infection. The order in which these virulence factors are expressed is important as bacterial pathogens traverse multiple microenvironments enroute to the final site of infection that can have varying pH, oxygen levels, or nutrient sources and must rapidly sense and adapt to these changes. Pathogenic *Escherichia coli* also need to time the expression of toxins, colonization factors, and secretion systems as these molecules are immunostimulatory and incur a high energy cost (Miao et al., 2010; Lee and Rietsch, 2015). Expression of these virulence factors are regulated at a transcriptional level (Bustamante et al., 2001; Lee et al., 2012; Thomas and Wigneshweraraj, 2014; Crofts et al., 2018; Connolly et al., 2019) however, post-transcriptional regulation by small RNAs, RNA-binding proteins (RBPs) and ribonucleases are equally critical in modulating gene expression of stress response genes and virulence factors in response to rapid changes in the host environment. Each of these RNA regulators are described in more detail below.

Regulatory RNAs, in particular *trans-*encoded small RNAs (sRNAs), serve important regulatory roles across all levels of gene expression. These include well-established mechanisms of regulating translation initiation and transcript stability (Waters and Storz, 2009; Papenfort and Vogel, 2010; Wagner and Romby, 2015). Since their initial discovery from intergenic regions of genes however, a slew of novel biogenesis pathways and regulatory mechanisms for sRNAs have been uncovered. sRNAs have since been found to originate from 3’ UTRs (Chao et al., 2012; Chao and Vogel, 2016; De Mets et al., 2019; Hoyos et al., 2020; Wang et al., 2020), 5’UTRs (Vanderpool and Gottesman, 2004; Thomason et al., 2019), protein-coding transcripts (Dar and Sorek, 2018), and pre-tRNAs (Lalaouna et al., 2015), and can effect regulation of their targets by modulating Rho-dependent termination (Sedlyarova et al., 2016; Silva et al., 2019; Hoyos et al., 2020), ribosome loading (Jagodnik et al., 2017; Romilly et al., 2019) and sponging interactions (Tree et al., 2014; Lalaouna et al., 2015; Miyakoshi et al., 2015)

Bacterial post-transcriptional regulation is also significantly influenced by global RNA-binding proteins (RBPs). In Gram negative bacteria for example, major post-transcriptional regulators include the conserved RNA-binding protein CsrA, and the RNA-chaperones such as Hfq and ProQ, which facilitate sRNA interactions with their targets (Vogel and Luisi, 2011; Romeo et al., 2013; Smirnov et al., 2016). Deletion of any of these RBPs has pleiotropic effects on gene regulation and pathogenicity (Timmermans and Van Melderen, 2009; Chao and Vogel, 2010; Vogel and Luisi, 2011; Potts et al., 2017; Westermann et al., 2019). Each RBP controls a large network of transcripts and may have antagonistic or overlapping roles, making for a sophisticated post-transcriptional network (Papenfort and Vogel, 2014; Melamed et al., 2020).

In this review, we discuss recent findings on sRNA regulation of virulence in the diarrheagenic *Escherichia coli* pathotypes EHEC, EPEC, and DAEC, and extraintestinal pathotypes UPEC and NMEC. In addition, we highlight how post-transcriptional regulation creates an additional layer of information that co-ordinates virulence factors, responds to environmental signals, provides increased stress tolerance and niche adaption, and even integrates host cell contact into virulence gene expression.

## Shiga Toxins and Phage Encoded Regulatory Small RNAs

Bacteriophages are major drivers of bacterial evolution and appear to provide a ready source of virulence genes that can augment bacterial virulence in a “plug and play” manner (Tobe et al., 2006; Loukiadis et al., 2008). In EHEC the primary cause of morbidity is the release of Shiga toxins (Stx) encoded within lambdoid bacteriophages. The Shiga toxins are AB_5_ toxins that bind to globotriaosylceramide (Gb3) that is expressed on renal epithelial cells and neurons, and causes cell death by depurinating and inactivating ribosomes. Shiga toxins are divided into two major groups, Stx1 and Stx2, that are further sub-divided into three subtypes of Stx1 (a, c, and d), and eight subtypes of Stx2 (a-h) (Scheutz et al., 2012; Bai et al., 2018). While multiple subtypes have been associated with human disease, Stx1a, Stx2a and Stx2d are associated with more severe disease outcomes (Melton-Celsa, 2014; Ogura et al., 2015). Three regulatory pathways that control expression of the Shiga toxins have been identified to date. The Shiga toxins are integrated into the late region of the Stx phage and in 2001 it was shown that transcription of the *stxAB* genes is driven by the Stx phage late promoter P_R’_ (Wagner et al., 2001; Wagner et al., 2002). The late promoter is regulated by phage induction and the RecA-dependent SOS response. Almost all of the identified regulatory signals that affect Stx production act through this pathway by modulating the SOS stress response. This pathway provides a single regulatory conduit from the Shiga toxin genes to quorum sensing, antibiotic stimulation, and small molecule inhibitors (Bielaszewska et al., 2012; Pacheco and Sperandio, 2012; Huerta-Uribe et al., 2016). Stx1 expression also responds to iron availability and nitric oxide stress through a second pathway. Early work had shown that the *stx1AB* genes were regulated by an upstream Fur-responsive promoter (P*_stx_*) that controls expression in response to iron and nitric oxide (Calderwood and Mekalanos, 1987). In addition, it is clear that a third regulatory pathway controls the Shiga toxins post-transcriptionally.

Analysis of Hfq binding sites in EHEC recovered extensive interactions with the Stx phages, including 11 intergenic peaks that were predicted to be regulatory small RNAs (Tree et al., 2014). Among the sRNAs identified, a Hfq-binding sRNA was produced from the region bounded by the late promoter P_R’_ and t_R’_. This region is constitutively transcribed and terminated during lysogeny as a by-product of antitermination regulation of the late promoter. Surprisingly, this short transcript is not simply degraded by the cell but is processed by RNase E to produce the stable sRNA termed StxS. StxS is expressed from both lysogenic Stx1 and Stx2a bacteriophages, and represses Shiga toxin 1 production 3-fold under lysogenic conditions by directly binding to *stx1B* RBS to silence translation (Sy et al., 2020). StxS does not appear to regulate *stx2AB* (that is only produced during lytic induction), although Stx2a is also likely regulated by as yet unidentified Hfq-dependent sRNAs (discussed below). StxS regulation of *stx1B* appears to provide a post-transcriptional check that suppresses Stx1 expression during lysogeny.

StxS sRNA also activates the stationary phase general stress response regulator RpoS through interactions with an activating seed region in the *rpoS* 5’ UTR (Sy et al., 2020). StxS binds to *rpoS* at the same site as the other known *rpoS-*activating sRNAs ArcZ, DsrA, and RprA (Lease et al., 1998; Majdalani et al., 2001; Mandin and Gottesman, 2010). These sRNAs unfold repressive secondary structure in the *rpoS* 5’UTR and inhibit premature Rho termination (Lease and Woodson, 2004; Updegrove et al., 2008; Soper et al., 2010; Sedlyarova et al., 2016). StxS likely acts through the same mechanism to constitutively activate *rpoS*, at least partly uncoupling *rpoS* from post-transcriptional repression in EHEC. Through StxS activation of *rpoS* translation, it was shown that EHEC is able to increase stationary phase cell density ~20% in nutrient limited minimal media (Sy et al., 2020).

Deletion of *hfq* in the EHEC strains 86-24 and EDL933 results in increased expression of *stx2AB*, suggesting that there are likely undiscovered sRNAs that can regulate expression of *stx2AB*, either directly or through modulation of the SOS response (Kendall et al., 2011). The Qin phage sRNA DicF has expanded from 1 to 4 copies in many EHEC strains and represses expression of *stx2A* (Melson and Kendall, 2019). DicF is upregulated during oxygen-limitation and this sRNA may link *stx2A* expression to oxygen availability during colonization, although the mechanism of DicF regulation of *stx2A* remains to be determined.

**Figure 1:**
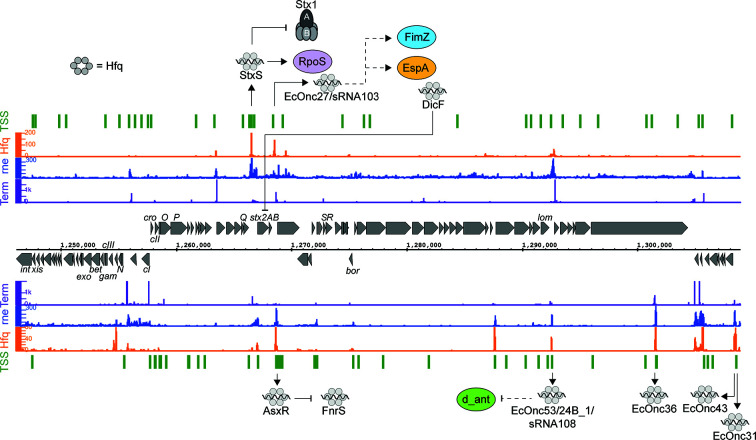
Post-transcriptional regulation to and from the Shiga toxin 2 phage. RNA sequencing data for the positive (top) and negative (bottom) strands are shown. Transcription start sites identified using differential RNA-seq (dRNA-seq) are indicated in green (GEO accession GSE143631). RNA 3’ends identified using Term-seq are indicated in purple. Hfq and RNase E-binding data from UV-crosslinking and sequencing experiments are shown in orange and blue, respectively (GEO accession GSE46118 and GSE77463). Regulatory RNAs encoded by the phage are indicated using outward arrows. Direct targets are indicated using solid line arrows, while dashed lines show indirect or unknown mechanisms of action.

Another sRNA has been identified within the late region of the Stx phage ϕ24_B_ and has been termed sRNA 24B_1. This RNA is unusual in that the mature sRNA is proposed to be processed from an 80 nt stem into a 20 nt ‘microRNA-sized’ regulatory RNA (Nejman-Faleńczyk et al., 2015). The authors identified this 20 nt fragment by selectively sequencing 10–40 nt RNAs present in ϕ24_B_ lysogenized commensal *Escherichia coli* MG1655. However, the mechanism of maturation, and function outside of the context of the longer 80 nt sRNA has not been determined. It remains likely that this 20 nt RNA represents a degradation product of the longer functional sRNA. In earlier analysis of Hfq binding sites in EHEC str. Sakai (Tree et al., 2014) this region of the Sp5 Stx2 phage was found to be bound by Hfq (termed sRNA EcOnc53). In our recent transcriptome-wide 5’ and 3’ end mapping data (dRNA-seq and Term-seq; Sy et al., 2020) we find that EcOnc53 is 67 nt in length (**Figure 1**). Gruber and Sperandio (2015) also identified EcOnc53 in the Stx phage BP933W, termed sRNA108 in their study. These authors found that sRNA108 was not destabilized by *hfq* deletion in EHEC str. EDL933. Extrapolating from experiments using the 189 nt ϕ24_B_ Δ24B_1 deletion and 566 nt complementation construct (Nejman-Faleńczyk et al., 2015), is seems that EcOnc53 may control the rate of phage lysogeny through interactions with Stx phage transcripts however, the mechanism of regulation remains unclear.

In addition to StxS and EcOnc53, ten additional Hfq binding sRNAs were identified in the Sp15 and Sp5 Stx phages (**Figure 1** and **Table 1**). Notably the *stxAB* genes are bookended by Hfq-binding sRNAs: StxS at the 5’ end and a dyad of sRNAs termed AsxR and EcOnc27 at the 3’ end (Tree et al., 2014). AsxR is a sRNA sponge that regulates that activity of the core genome-encoded sRNA FnrS and relieves repression of the haem oxygenase *chuS* involved in haem uptake (Tree et al., 2014). EcOnc27 was also identified in EHEC str. EDL933 and termed sRNA103 (Gruber and Sperandio, 2015). Overexpression of sRNA103 was found to upregulate *fimZ* although the mechanism of regulation is not clear.

**Table 1:** Hfq-dependent sRNAs identified in the Shiga toxin-encoding bacteriophages.

Stx phage	sRNA	Synonym	Experimental confirmation	Targets	Direct interaction?	References
**Stx2Φ**	AsxR	EcOnc02	Northern probing; Hfq-CRAC	FnrS	Yes	Tree et al., 2014
	StxS	EcOnc15	Northern probing; Hfq-CRAC	*rpoS*	Yes	Tree et al., 2014
				*stx1b*	Yes	Sy et al., 2020
	EcOnc27	sRNA103	Northern probing; Hfq-CRAC			Tree et al., 2014
				*fimz;espA*	*In silico* yes; *In silico* no	Gruber and Sperandio, 2015
	EcOnc53	sRNA108; 24B_1	Northern probing; Hfq-CRAC	*d_ant*	*In silico* yes	Tree et al., 2014; Nejman-Faleńczyk et al., 2015
	sRNA110	none	Northern probing	unknown	N/A	Gruber and Sperandio, 2015
	EcOnc20	none	Hfq-CRAC	unknown	N/A	Tree et al., 2014
	EcOnc50	none	Northern probing; Hfq-CRAC	unknown	N/A	Tree et al., 2014
	EcOnc36	none	Hfq-CRAC	unknown	N/A	Tree et al., 2014
	EcOnc43	none	Hfq-CRAC	unknown	N/A	Tree et al., 2014
	EcOnc31	none	Hfq-CRAC	unknown	N/A	Tree et al., 2014
**Stx1Φ**	EcOnc22	EcOnc23; EcOnc24	Northern probing; Hfq-CRAC	unknown	N/A	Tree et al., 2014
	EcOnc42	EcOnc21; EcOnc08	Northern probing; Hfq-CRAC	unknown	N/A	Tree et al., 2014

Collectively, the repertoire of regulatory sRNAs encoded within the Stx phages have been shown to directly control Shiga toxin 1 expression and growth, and at least indirectly control lysogeny and *fimZ* expression. Oxygen-limitation also appears to be an important signal as both DicF (represses *stx2A*) and FnrS (repressed by AsxR) are induced under this condition. Given that tissue damage from the toxin may release haem, the logic behind this regulatory circuit is not clear, but it seems that haem uptake is promoted by the Stx phage (AsxR) while the core genome acts to repress Stx2 toxin expression under oxygen limitation. Further investigation will likely reveal the selective pressures that drive maintenance of these regulatory pathways.

*Escherichia coli* prophages appear to be littered with regulatory sRNA and some have been shown to modulate virulence. An additional oxygen-sensitive sRNA was recently described in the neonatal meningitis causing *Escherichia coli* (NMEC) strain K1. For NMEC to establish infection in the central nervous system, it must survive in the microaerophilic environment of blood and cross the blood brain barrier (Doran et al., 2016). In this low oxygen environment, the transcription factor ArcA inhibits expression of the mEp460 phage-encoded sRNA, sRNA-17. Using a mouse model of meningitis, it was shown that deleting this sRNA results in increased survival in blood and improved penetration of the blood brain barrier (Song et al., 2020). While the direct targets of this sRNA have not been identified, the authors propose that this phenotype is a result of metabolic changes during growth in blood.

The small RNA sponge, AgvB, is also encoded within a cryptic EHEC prophage and represses the sRNA GcvB (Tree et al., 2014). GcvB represses amino acid uptake pathways and AgvB de-represses these transporters through interactions with the GcvB R1 seed region. AgvB allows increased growth in bovine terminal rectal mucus (the reservoir host for EHEC) demonstrating that horizontal acquisition of sRNA sponges can adapt core genome-encoded regulatory pathways for fitness in a specialized niche. AgvB (like AsxR and EcOnc27) is encoded within a dyad of sRNAs, in this case AgvB is transcribed from the antisense strand of EcOnc06. These dyads of Hfq-binding sRNAs are positioned between the lysis genes and late promoter of at least eight lambdoid phages in EHEC str. Sakai (Tree et al., 2014). Why this arrangement of sRNAs occurs is unclear, but it appears to be a source of regulatory information that adapts EHEC to a pathogenic lifestyle.

## Colonization and Adhesion

### Type 3 Secretion in Attaching and Effacing Pathotypes

Both EPEC and EHEC are enteric pathogens that cause moderate to severe levels of diarrhea. Diarrheal disease is dependent on formation of attaching and effacing (A/E) lesions on the intestinal epithelia, which is conferred by genes encoded within the locus of enterocyte effacement (LEE). The LEE encodes a type 3 secretion system (T3SS) that injects a cocktail of bacterial effectors that affect cellular processes such as modification of the actin cytoskeleton, disruption of microtubule networks, and ion uptake, which collectively result in the formation of an actin pedestal at the site of attachment that is characteristic of EPEC and EHEC (Galán et al., 2014; Wagner et al., 2018).

**Figure 2:**
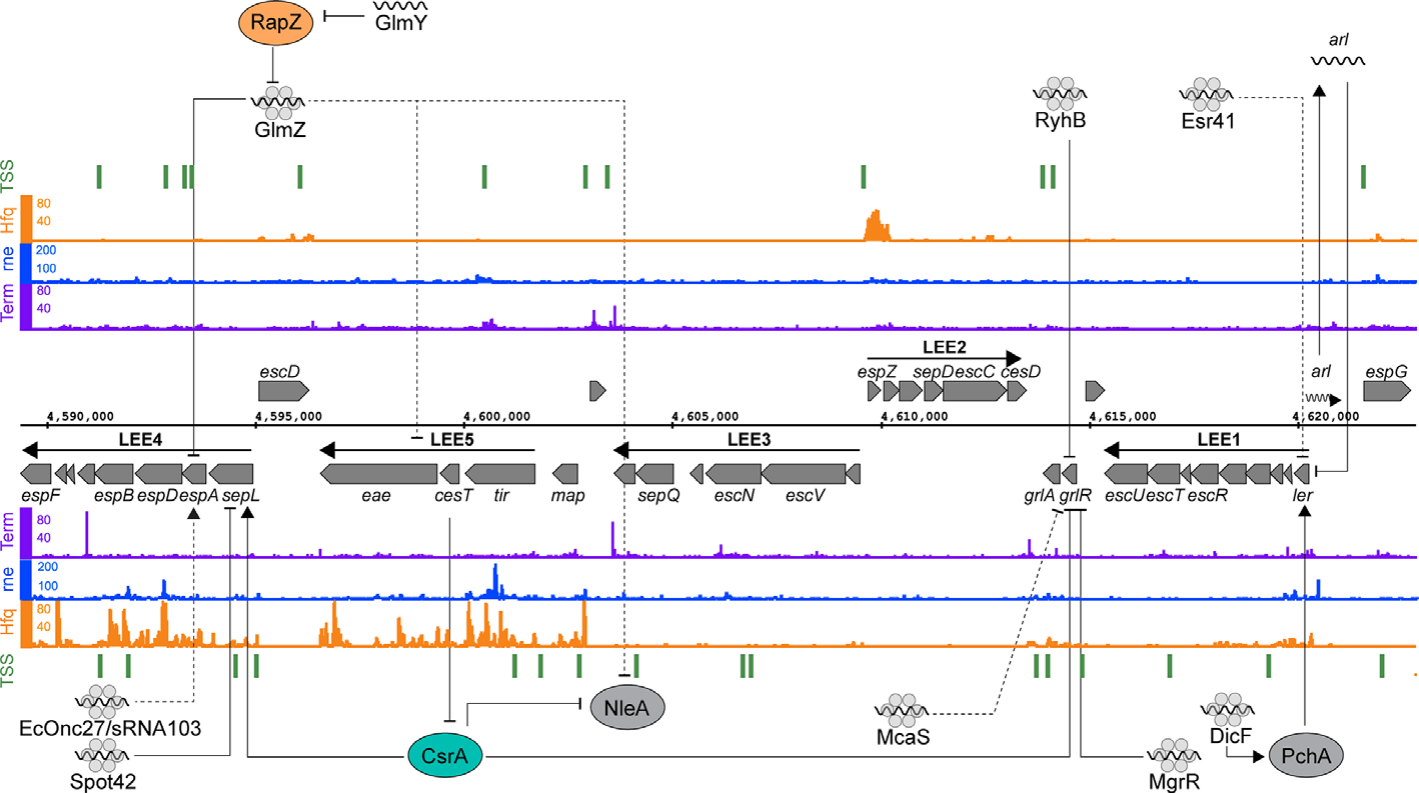
Post-transcriptional regulation of the locus of enterocyte effacement. RNA sequencing data for the positive (top) and negative (bottom) strands are shown. Transcription start sites identified using differential RNA-seq (dRNA-seq) are indicated in green (GEO accession GSE143631). RNA 3’ends identified using Term-seq are indicated in purple (GEO accession GSE14363). Hfq and RNase E-binding data from UV-crosslinking and sequencing experiments are shown in orange and blue, respectively (GEO accession GSE46118 and GSE77463). Direct targets for post-transcriptional regulators are pointed to by solid black arrows, while indirect targets are indicated with dashed lines.

The LEE consists of five major operons (LEE 1-5) (**Figure 2**) and is subject to extensive post-transcriptional regulation that coordinates expression within operons and with external loci. Transcriptional regulation is controlled by the master regulators Ler and GrlRA, acting in concert with a panoply of transcription factors that integrate environmental signals that indicate host infection (Tree et al., 2009; Turner et al., 2018). The polycistronic transcripts of the LEE are regulated by the global RNA binding proteins CsrA and Hfq. Deletion of *hfq* upregulates LEE expression in strains Sakai and EDL933, but paradoxically represses T3S in strain 86-24 (Hansen & Kaper, 2009; Shakhnovich et al., 2009; Kendall et al., 2011; Wang et al., 2017; Wang et al., 2018). In EDL933, Hfq represses *grlA* expression during exponential phase, which indirectly represses the expression of Ler (Hansen and Kaper, 2009). In stationary phase Hfq appears to be able to directly repress the LEE in an *ler-*dependent, GrlA-independent manner (Shakhnovich et al., 2009). In the EHEC strain 86-24, Hfq is reported to activate expression from the LEE (Kendall et al., 2011). This effect has been attributed to the differing sRNA repertoires in mosaic EHEC genomes. Indeed, copy numbers of EHEC-specific sRNAs such as StxS, EcOnc10 and EcOnc42 appear to vary between EHEC strains Sakai and 86-24.

Both pathotype-specific and core genome-encoded sRNAs control expression of the T3SS. The EHEC specific sRNA Esr41 represses *ler* although the mechanism of repression is yet to be identified (Sudo et al., 2018). In EPEC, the core genome encoded sRNAs MgrR and RyhB regulate expression of the LEE by directly base-pairing with different regions of the *grlRA* transcript (Bhatt et al., 2017). Base-pairing of RyhB close to the ribosome binding site of *grlRA* results in the repression of the entire operon, resulting in LEE repression. MgrR on the other hand binds close to the transcription start site and appears to repress *grlR* alone. The repression of *grlR* prevents it from regulating *grlA*, resulting in activation of the latter. RyhB and MgrR are regulated by iron and magnesium availability respectively, suggesting that metal ion availability plays an important role in post-transcriptional activation and repression.

Transcription of the master regulator *ler* is induced by the non-LEE encoded transcriptional regulators PchABC. Under microaerobic conditions, such as those experienced in the gastrointestinal tract, the sRNA DicF activates translation of *pchA* by disrupting a secondary structure formed by the *pchA* leader region that blocks ribosome association (Balasubramanian et al., 2016; Melson and Kendall, 2019). PchA then activates transcription of *ler*, which results in increased LEE expression (Iyoda and Watanabe, 2004; Melson and Kendall, 2019). This represents another example of a core genome encoded sRNA being co-opted by *Escherichia coli* pathotypes to regulate virulence genes. As mentioned earlier, this regulation appears to have been expanded in EHEC strains Sakai and 86-24 that have four copies of *dicF*, while the commensal *Escherichia coli* encodes a single copy in the cryptic Qin prophage.

In EHEC strains, translation of the T3SS filament EspA from LEE4 is heterogenous at the single cell level despite homogenous transcription levels among the bacterial population (Roe et al., 2003; Roe et al., 2004). While the mechanism of heterogeneity has yet to be uncovered it is clear that the LEE4 operon is subject to quite sophisticated post-transcriptional regulation. SepL is encoded at the start of the polycistronic LEE4 operon and acts as a gate keeper to the T3S translocon (O’Connell et al., 2004; Deng et al., 2005). The *sepL* 5’UTR adopts a translationally inactive “clover leaf” structure consisting of four stem loops that occlude the RBS of the nascent mRNA (Wang et al., 2018). The global RNA binding protein CsrA activates LEE4 by directly binding to the leader region of the *sepL-espADB* transcript to unfold the repressive cloverleaf structure and allow translation of *sepL* (Bhatt et al., 2009; Wang et al., 2018). The open structure is then a target for Hfq and the sRNA Spot42 that repress translation of *sepL* through direct base-pairing with the RBS. The binding constant (K_d_) for CsrA-*sepL* is 23 nM (Bhatt et al., 2009) and Hfq interactions with mRNAs typically have a K_d_ of 1-4 nM (Fender et al., 2010) suggesting that the nascent *sepL* transcript would rapidly toggle from translationally OFF to ON to OFF after transcription. Spot42 and CsrA are regulated by the availability of preferred carbon sources and their relative concentrations would likely be in lock step, maintaining competition between the regulators to rapidly toggle between OFF-ON-OFF states (Wang et al., 2018). The effect of this post-transcriptional toggle is expected to be a limited round of *sepL* translation. Importantly, the downstream *espADB* operon is cleaved from the *sepL-espADB* transcript (Lodato and Kaper, 2009) and can undergo independent translation. SepL is required at a lower stoichiometry than the EspADB needle filament and tip proteins, and the limited burst of *sepL* translation likely contributes to producing the correct stoichiometric ratio of SepL to EspADB for T3SS assembly.

Expression of the LEE4-encoded *espADB* and transcripts from LEE5 are also repressed by the paralogous sRNAs GlmY and GlmZ (Gruber and Sperandio, 2014; Gruber and Sperandio, 2015). The effects of GlmY on the LEE is due to its established role of sequestering the RNase E adapter protein RapZ from GlmZ (Göpel et al., 2013; Gruber and Sperandio, 2015). GlmZ repression of LEE5 is due to an unknown indirect mechanism, while regulation of *espADB* is due to direct binding of GlmZ to the 3’ region of LEE4. GlmZ binding to the *espADB* transcript results in its destabilization (Gruber and Sperandio, 2014). While recruitment of RNase E to the GlmZ-LEE4 binding site has been ruled out as a mechanism for destabilization of the *espADB* transcript, other explanations could include processing by RNase III, or possibly recruitment of Rho.

CsrA binds to AUGGA sequence motifs in stem loops to both positively and negatively regulate translation (Romeo et al., 2013; Holmqvist et al., 2016; Potts et al., 2017). In addition to LEE4, CsrA controls a broad range of bacterial processes including central carbon metabolism, motility and virulence (Romeo et al., 2013; Vakulskas et al., 2015). In EPEC and EHEC, CsrA has opposing regulatory effects on *sepL* (activating) and *grlRA* (repressive) suggesting temporal separation of these events (Wang et al., 2017; Wang et al., 2018). CsrA also represses translation of the non-LEE encoded effector *nleA*, which is expressed after attachment to host cells, and controls host inflammatory pathways (Gruenheid et al., 2004; Kim et al., 2007; Yen et al., 2015). The leader region of the *nleA* transcript contains two CsrA binding sites, and translation of this *nleA* is repressed by CsrA binding (Katsowich et al., 2017). In EPEC, CsrA activity is modulated by the T3SS effector chaperone CesT, which interacts with Tir and other effectors (Abe et al., 2002; Katsowich et al., 2017). Importantly, Tir is the first effector secreted after host cell contact, releasing CesT. Free CesT binds the mRNA-binding surface of CsrA, resulting in its sequestration and de-repression of *nleA* translation (Ye et al., 2018). This interaction provides an elegant mechanism to couple host cell contact to translation of secreted effectors. Sequestration of CsrA also modulates bacterial carbon metabolism to suit an A/E lifestyle.

The LEE-encoded T3SS is a complex molecular machine regulated at the transcriptional, post-transcriptional, and post-translational level to ensure appropriate gene expression in response to environmental cues, subunit stoichiometry, and temporal signals during T3S assemble and host cell contact. It is clear that CsrA, Hfq, and regulatory sRNAs play critical roles in regulating these processes and we expect that many more RNA-based regulatory signals that control elaboration of this sophisticated machine remain to be discovered.

### Regulation of Fimbriae and Invasins

UPEC are the major causative agent of uncomplicated urinary tract infections (UTI). This pathotype colonizes the bladder, causing cystitis, that can lead to ascending infections of the kidney termed pyelonephritis. Its success as a pathogen is due to a wide array of virulence factors, such as toxins, iron-acquisition systems, flagella, fimbrial adhesins and other surface structures (Terlizzi et al., 2017). Fimbriae are essential for attachment to host cells and the formation of intracellular bacterial communities (IBCs) (Subashchandrabose and Mobley, 2015). Type 1 fimbriae bind uroplakins on the surface of bladder epithelial cells (Wu et al., 1996; Chahales and Thanassi, 2015) and P-fimbriae are required for colonization of the kidney and progression to pyelonephritis (Roberts et al., 1994; Dodson et al., 2001; Chahales and Thanassi, 2015).

The RNA chaperone Hfq is required for UPEC pathogenicity and it was posited that this was exerted through sRNAs regulation of outer membrane homeostasis (Kulesus et al., 2008). Hfq RIP-seq was used to identify sRNAs expressed during UPEC infection of epithelial cells and in liquid culture. Interestingly, this study identified C271 as a UPEC-specific sRNA expressed in both conditions, though the role of C271 in pathogenesis was not explored further. Another sRNA, PapR, which can also be found in some EHEC and *Shigella* species, was found to be more highly expressed during host cell infection (Khandige et al., 2015). This sRNA was found to repress expression of the P-fimbriae regulator *papI*, which is required for phase-variation of this virulence factor. PapR achieves this repression by binding +74 to +96 nucleotides downstream of the translational start site, suggesting that it may regulate *papI* by recruiting RNases, or by affecting ribosomal loading (Bandyra et al., 2012; Jagodnik et al., 2017).

An *in silico-*based screen in UPEC strain 536 and ExPEC strain AL862 identified 5 sRNAs expressed antisense to the coding region of virulence genes. Three of these sRNAs, PrfR, HlyR, and HaeR were predicted to interact with the mRNAs *prfF, hlyR*, and *haeR*, respectively. One sRNA discovered using this screen was FimR, which is encoded antisense to the 3’ end of the fimbrial usher gene *fimD.* FimR regulates type 1 fimbriae gene expression by directly activating *fimD* (Pichon et al., 2012). Changes in *fimD* expression were not observed in a Δ*hfq* mutant carrying a plasmid encoding FimR, suggesting that this interaction is Hfq-dependent (Pichon et al., 2012). However, Khandige et al. (2015) note that FimR is not detected in their Hfq co-IP study performed in UPEC strain UTI89 despite the conservation of the *fim* operon between strains (Khandige et al., 2015). These divergent results may suggest that the regulation of *fimD* by FimR is not directly mediated by Hfq. As such, additional experimental verification is required to understand whether Hfq is needed for FimR base-pairing with *fimD.*

AfaR is a sRNA expressed by pathogenic *E. coli* that harbor the *afa-8* PAI, which includes some DAEC and ExPEC strains (Lalioui and Le Bouguénec, 2001; Pichon et al., 2013; Servin, 2014). AfaR is encoded antisense to the intergenic region of *afaD* and *afaE* invasins and is regulated by both temperature and by an RpoE-dependent promoter. AfaR regulates expression of AfaD-VIII invasins by binding to the 5’UTR of *afaD* in an Hfq-dependent manner, and promotes its degradation by RNase E (Pichon et al., 2013). AfaR expression is repressed by temperatures higher that 37°C likely to allow for expression of the AfaD-VIII invasion once the pathogen has entered the host.

### Motility

Motility plays an important role in colonization through inducing and evading the host immune response, as well as movement to sites of infection and environments that are more suitable for bacterial growth (Josenhans and Suerbaum, 2002). In commensal *Escherichia coli*, expression of the master flagellar regulator FlhDC is subject to post-transcriptional regulation by CsrA and the sRNAs ArcZ and OxyS (Wei et al., 2001; De Lay and Gottesman, 2012; Thomason et al., 2012; Mika and Hengge, 2013; Yakhnin et al., 2013; Bak et al., 2015). In EHEC, motility is coordinated with expression of the T3SS during attachment (Iyoda et al., 2006). The EHEC specific sRNA Esr41 has been found to repress the LEE and increase motility through activation of *fliC* (Sudo et al., 2014; Sudo et al., 2018). However, while LEE repression appears to be is due to an interaction between the leader region of the *ler* mRNA and Esr41, no sites of complementarity between Esr41, *ler*, and *fliC* have been identified, and the mechanism of regulation is still unclear (Sudo et al., 2014; Sudo et al., 2018).

## Iron Homeostasis

Iron is essential for maintaining bacterial homeostasis but is also required for virulence of a variety of pathogens (Anzaldi and Skaar, 2010; Skaar, 2010). Vertebrate hosts prevent infection by depriving pathogens of this valuable cofactor through sequestration, termed nutritional immunity. Despite its utility, an excess of iron is detrimental due to the build-up of free radicals that can damage both DNA and the bacterial membrane (Anzaldi and Skaar, 2010). Bacteria have developed elegant ways to maintain iron homeostasis, and key among these is the transcriptional regulator Fur, and Fur-regulated sRNA RyhB (Braun and Braun, 2002; Massé et al., 2005; Massé et al., 2007; Chareyre and Mandin, 2018). In *Pseudomonas aeruginosa* the RyhB analog PrrF control the iron-sparing response and is required for virulence in a mouse model of lung colonization (Reinhart et al., 2015; Reinhart et al., 2017). In commensal *Escherichia coli*, siderophore production is regulated through RyhB interactions with *shiA* and *entCEBAH* (Prévost et al., 2007; Salvail et al., 2010). In UPEC, RyhB additionally positively regulates production of the pathotype-specific siderophores aerobactin and salmochelins. Deleting *ryhB* resulted in reduced colonization of the bladder and kidneys in a mouse model of infection, and down regulation of the aerobactin synthesis gene *iucD* indicating that RyhB and aerobactin are required for uropathogensis (Porcheron et al., 2014).

Bacterial pathogens can also acquire iron through haem acquisition and degradation systems (Runyen-Janecky, 2013; Choby and Skaar, 2016; Richard et al., 2019). In EPEC, EHEC and UPEC, a TonB-dependent outer membrane haem receptor is encoded by *chuA* (Torres and Payne, 1997; Nagy et al., 2001; Hagan and Mobley, 2009). The 5’UTR of *chuA* is unusually long at approximately 300 nucleotides (Nagy et al., 2001). In EHEC, sRNA interactome sequencing (RNase E-CLASH) revealed that *chuA* is repressed by the pathotype-specific sRNA Esr41 through direct base-pairing. The same study identified *bfr* (bacterioferritin) and *cirA* (catecholate siderophore receptor) as targets for Esr41 (Waters et al., 2017). Interestingly, these genes are also targets for RyhB, suggesting that Esr41 may be a pathotype-specific sRNA for regulating iron homeostasis (Massé and Gottesman, 2002; Salvail et al., 2013; Porcheron et al., 2014). Surprisingly, while RyhB activates translation of *cirA*, Esr41 was found to repress CirA expression. This difference may allow EHEC to preferentially use the adhesin and enterobactin siderophore Iha, which is encoded immediately upstream of Esr41, as an iron acquisition system (Waters et al., 2017).

Iron acquisition and nutritional immunity have long been known to play critical roles in infection and recent studies have demonstrate that post-transcriptional regulation of iron homeostasis is required for disease progression.

## Where to From Here?

It has become increasingly clear that post-transcriptional regulation is vital for regulation of virulence in pathogenic *Escherichia coli* and significant progress has been made to better understand these mechanisms. The advent of high-throughput approaches to map Hfq interactions, and identify sRNAs and their targets using MAPS, CRAC, CLASH, RIL-seq and GRIL-seq have provided a window into the extent of sRNA regulation (Lalaouna et al., 2015; Han et al., 2016; Melamed et al., 2016; Waters et al., 2017; Iosub et al., 2020a; Wong et al., 2018). In EHEC alone, 55 novel sRNAs were found along the pathogenicity islands of O157:H7 str. Sakai (Tree et al., 2014). However, the analysis used to identify these sRNAs searched for orphan Hfq-binding sites that were >100 nt from any annotated coding region. Recently, the 3’UTR of coding genes have emerged as a significant source of regulatory sRNAs, and have been found to regulate the outer membrane stress response, nitrate transport, and acetate and carbon metabolism (Chao et al., 2012; Chao and Vogel, 2016; De Mets et al., 2019; Hoyos et al., 2020; Iosub et al., 2020b; Wang et al., 2020). Taken together, this suggests that there may be many more pathotype-specific sRNAs than we currently appreciate, and it will be intriguing to investigate how these 3’UTR, 5’UTR, and recently identified CDS-encoded sRNAs may affect bacterial virulence.

RNA binding proteins play important roles in facilitating sRNA interactions and methods for discovering novel RNA-binding proteins will undoubtably provide new insight into post-transcriptional regulation of bacterial virulence. Indeed, the development of Grad-seq in *Salmonella* led to the discovery of a second sRNA chaperone ProQ, which governs its own sRNA network distinct from Hfq and CsrA (Smirnov et al., 2016). ProQ is required for pathogenicity in *Salmonella enterica*, suggesting the same may be true for pathogenic *Escherichia coli*. An analysis of the ProQ RNA interactome in commensal *Escherichia coli* has shown that while ProQ has a distinct RNA interactome, it has some overlap with Hfq that provides additive or divergent effects (Melamed et al., 2020). Further investigation into the effects of ProQ in pathogenic *Escherichia coli* virulence is certainly warranted and may identify regulatory pathways required for virulence.

Additional methods for identifying the RNA-binding proteome have recently been developed for prokaryotes. These include TRAPP (Shchepachev et al., 2019), PTex (Urdaneta et al., 2019) and OOPS (Queiroz et al., 2019). The development of these methods provide exciting opportunities for discovering novel RNA-binding proteins that may have significant roles in infection and pathogenicity.

## Author Contributions

All authors listed have made a substantial, direct and intellectual contribution to the work, and approved it for publication.

## Funding

This work has been supported by an operating grant from the National Health and Medical Research Council (GNT1161161).

## Conflict of Interest

The authors declare that the research was conducted in the absence of any commercial or financial relationships that could be construed as a potential conflict of interest.
